# Stable isotopes in tissues discriminate the diet of free-living wild boar from different areas of central Italy

**DOI:** 10.1371/journal.pone.0183333

**Published:** 2017-08-17

**Authors:** Giuseppe Russo, Pier Paolo Danieli, Riccardo Primi, Andrea Amici, Marco Lauteri

**Affiliations:** 1 Institute of Agro-Environmental and Forest Biology (IBAF), National Research Council (CNR), Porano, Italy; 2 Department of Agricultural and Forestry Sciences (DAFNE), University of Tuscia, Viterbo, Italy; University of Hyogo, JAPAN

## Abstract

The use of isotopic signatures in animal tissues provides information on the environment where they are living and, notably, on their diet. Carbon and, whenever possible, nitrogen stable isotope analyses were performed in animal hairs, muscles and fat. Particularly, we analyzed both carbon and nitrogen isotopic compositions (δ^13^C and δ^15^N) on wild boar samples across three different areas of central Italy (Latium region): Tyrrhenian Coast (TC), Maremma (MA) and Central Plains (CP). The agricultural habits of these areas imply that, in winter, no crops are available for wild boars, which feed mainly on acorns and natural feeds (tubers, earthworms *etc*.). In addition, the three areas were influenced by oak masting. One of these areas (CP) was characterised by the spreading of corn during the hunting season to attract the animals. For each area, we sampled 10 animals aged between 12 and 24 months and balanced by gender. Anenrichment of δ^13^C in CP area, where corn was used, was observed in all the analysed tissues in comparison to other areas (MA and TC). In CP area, enriched values of δ^15^N were also observed in all the tissues. The research demonstrates that both δ^13^C andδ^15^N in free-living wild boar tissues are influenced by sampling area. According to feeding habits of the species and wildlife management (feed supplementation), the differences observed in δ^13^C and δ^15^Nare based on the specific feeding regime; particularly the use of corn in wintertime. Furthermore, the research highlights and discusses diversities and relationships among δ^13^C and δ^15^N in the hair, fat and muscles of free-living wild boar.

## Introduction

Stable isotopes are naturally present in animal tissue and reflect the isotopic composition of diet [1]; carbon and nitrogen isotopic compositions (δ^13^C and δ^15^N) are influenced by feeding practices and climate [2]. This is because the isotopic fractionations occurring along the primary productivity processes (see Brugnoli and Farquhar, 2000) [3] and along the nitrogen cycle (see Amundson et al., 2003) [4] affect both δ^13^C and δ^15^N of the plant material that is at the beginning of the trophic chain. Certain environmental and biological processes may operate selectively in favour of either the heavier or the lighter stable isotopes [5]. Such isotopic effects of fractionation on the primary sources of feeding are modified by the animals’ metabolic processes and finally incorporated into the animal tissues, determining their isotopic signatures [6]. For example, animal hair represents an archive of the isotopic signatures of food eaten by the animal at the time the hair was laid down, recovering information on the animal’s environment and feeding habits [7]. Muscle is one of the main tissues used for determining the stable isotope signature of animals [8]. Furthermore, stable isotope ratio analysis on skeletal muscle has been used as a tool to authenticate meats from beef cattle by the quantification of isotopic turnover of C and N [1,9].

DNA-based technology allows animal identification but does not provide information about feed history or the production system under which the animal was grown. On the contrary, stable isotope methodologies are particularly appropriate in describing a specific position within a food web and in investigating the trophic relationships of a species. Stable isotopes of light elements, those primarily involved in many biological and biogeochemical processes, are particularly informative. In fact, the isotopic fractionations occurring along all the biogeochemical cycles represent powerful tracers of the cycles themselves. The common way to say “you are what you eat” is especially true from a stable isotope perspective. The primary productivity processes driven by the photosynthesis generate well distinguished isotopic pools of organic carbon owing to a number of biophysical fractionations, which are affected by complex environmental and genetic determinants. For instance, the photosynthetic metabolism strongly determines the overall isotope composition of plant carbon. Owing to the different isotopic fractionations operated by the carboxylases Rubisco and PEPcase and to the different biophysical paths for the CO_2_ molecules, C3 plants are much more depleted in ^13^C rather than C4 plants [3]. Furthermore, in fresh and humid or irrigated ecosystems, C3 plants usually show more depleted δ^13^C than in drought prone environments. This is explained by the resultant of the isotopic fractionations occurring both along the diffusional path of the CO_2_ from the outer to the interior of the leaf and along the biochemical reactions of carboxylation in the chloroplasts [10]. The ecosystem nitrogen cycle is also characterized by a complex of isotope fractionations occurring along the chemical transformations, which link the different N pools. For instance, anthropogenic N pulses are usually depleted in ^15^N in respect to the isotopic abundance of the N pools in the natural ecosystem biomass. Processes of mineralization, nitrification and ammonification, in interaction with the soil biota, cause important fractionation phenomena, favoring the isotopic diversification of the N sources entering the food web. Thus, using a multi-isotope approach in analysing animal tissues can reveal their origin by unravelling the complexity of the environmental characteristics and local land uses [11]. Such a complexity, finally, is reflected across the local food web. In fact, the δ^13^C value of the whole animal body reflects the food consumed, although enriched in ^13^C by about 1‰ respectively to the feeding source. The corresponding δ^15^N values appear more variable but are enriched, on average, about 3‰ [12]. Several studies investigated cases about animals and mammals in particular [5,9,12]. The results generally imply an isotopic matching with the prevalent C3 or C4 plant source in the diet. The enrichment in ^15^N of the animal tissues reflects fractionations occurring during the N metabolism and the protein synthesis [12].

The wild boar (*Sus scrofa*) is the only non-ruminant ungulate with a three-glandular stomach, similar to mammals. It is an omnivorous species and its diet depends on the available food resources: in winter, vegetables (acorns, roots, tubers, chestnuts) represent most of the total food mass (80–90%), while food of animal origin, such as arthropod larvae, annelids, micro-mammals or remains is consumed throughout the year but mainly in winter [7,13]; considering the crops, corn (*Zea mays*) is an important and dominant part of the diet for wild boar in Europe [14,15].

Determinations of stable isotope ratios of carbon (^13^C/^12^C) and nitrogen (^15^N/^14^N) in organic matter can be used to reconstruct dietary patterns and to investigate the environmental conditions existing at the time of tissue formation [8,16].

As the use of supplementary feeds is a relevant and wide spread tool in wild boar management, the issues explored in this work are: a) assessing whether carbon (δ^13^C)and nitrogen (δ^15^N) isotope compositions in free-living wild boar tissues are affected by sampling area; b) to attempt the interpretation of feeding regime on the basis of stable isotope assays; c) to explore tissue diversity and relationships concerning isotope compositions.

## Material and methods

### Sampling areas, land use and climate

**Fig 1:**
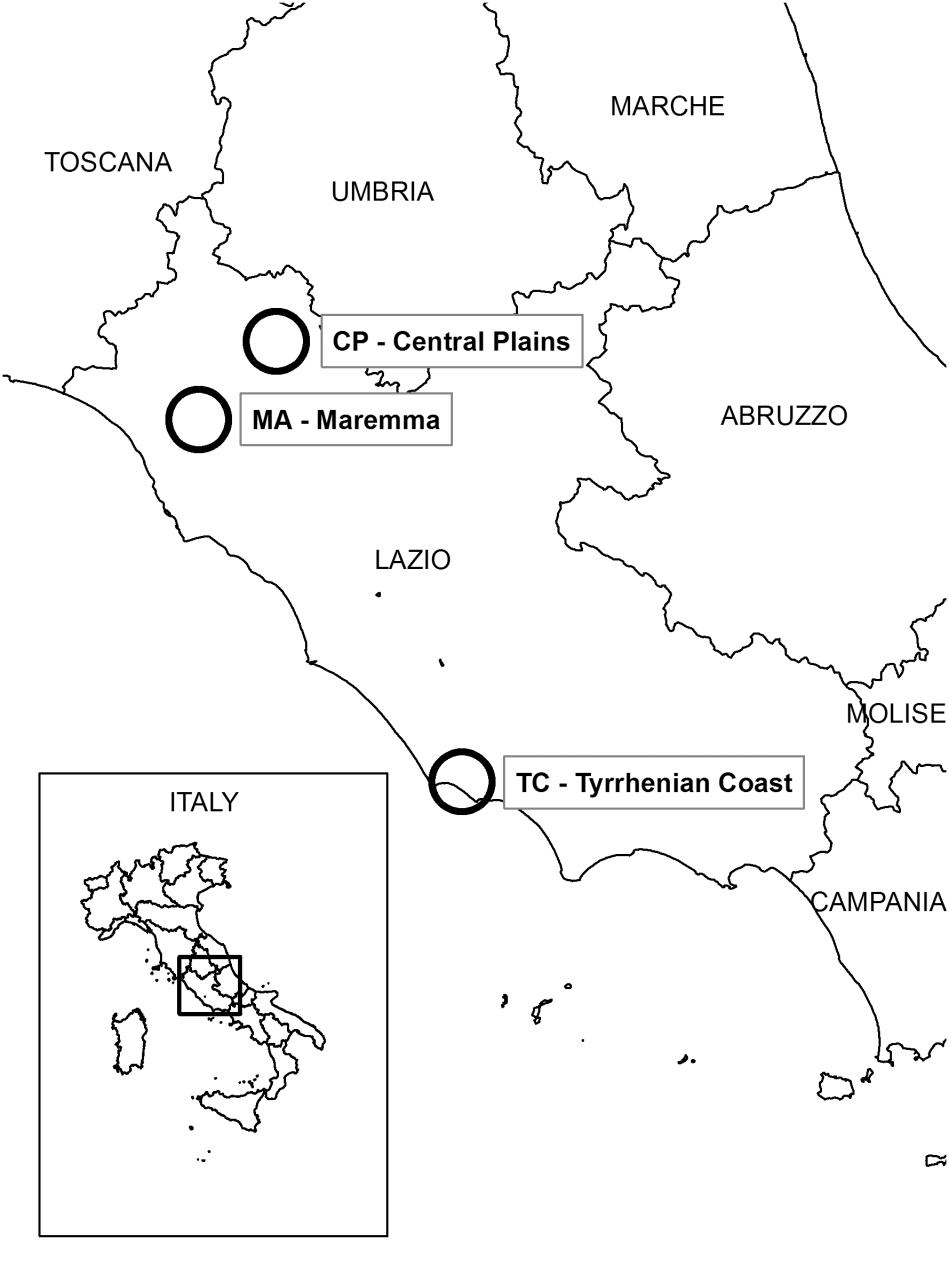
Sampling areas (TC: Tyrrhenian Coast, MA: Maremma, CP: Central plains).

Animals were sampled in three areas of Central Italy (Fig 1): Tyrrhenian Coast (TC), Maremma (MA), and Central Plains (CP). Those areas are characterized by different land cover and wildlife management and, particularly, wild boar management. The land cover types were calculated on the basis of a circular area centered in the harvesting area covering about 100 km^2^. The Lazio Region land cover archive was used (CUS) [17]. The land cover classes are reported in Table 1 in order to highlight the main differences. TC is a flat area (81 m a.s.l.) characterized by moors and heathlands nearby the seaside and about 8% of woods mainly represented by coniferous plantations and broadleaf stands. Swamps and water bodies represent 4% of the surface. No wild boar feed supplementation was adopted. MA is a hilly area (184 m a.s.l.) mainly covered by agricultural land (80%) and about 20% of woods and natural surfaces. MA also has an unmanaged ban on hunting areas. No wild boar feed supplementation was adopted. CP is a flat area with some hills (112 m a.s.l.). It is covered by agricultural land (72%) with a wooded body covering about 27% of the surface. As private hunting groups manage the area, corn spreading to attract animals is performed throughout the hunting season, since November until January.

**Table 1:** Land cover (%) and climatic data (mean annual temperature, mean annual rainfall and xerothermic index) of the three areas (TC: Tyrrhenian Coast, MA: Maremma, CP: Central plains).

Land cover class	TC	CP	MA
Urban	0	1	1
Agriculture	1	72	80
Woods	8	25	17
Swamp	3	0	0
Water	1	0	0
Shrubs and natural areas	87	2	2
Climate			
Annual Mean T (°C)	15.6	16.3	16.6
Rainfall (mm)	1000	746	667
Xerothermic index	89.6	78.6	158.8

The agricultural habits of these areas imply that during winter no crops are available for wild boars, which feed mainly on acorns and natural feeds (tuber, earthworm *etc*.). In addition, the areas are influenced by recurrent oaks masting.

Climate data (Table 1) were obtained by the Servizio Integrato Agrometeorologico Regione Lazio (SIARL), recognizing the data related to the following stations: Tarquinia Portaccia (MA), Canino Pianacce (CP) and Roma Capocotta (TC). Xerothermic Index (X_i_) [18] for each site was calculated using the formula reported by Leone [19].

### Sampling procedures and sample preparation

Wild animals were not killed intentionally for the reasons of this research and, as such, no specific authorization was needed for tissue and organ sampling according to the applicable National laws. The study was carried out by using tissues and organs of free-living wild boar killed during regular hunting under applicable National laws. In all of the cases, sampling of organs and tissues were performed during the slaughtering and dressing procedures. Animals were sampled in January 2016 during the hunting season. The animals were harvested by local hunting teams, and were individually identified before carcass processing. Body weight and some morphological traits were measured before slaughtering (Table 2) according to [20]. Age was determined by teeth eruption and wear [21] during slaughtering. Samples of skin (shoulder), including hair, peri-renal fat and muscular tissue from the foreleg were also collected. Samples were identified and individually stored at 5°C. All the samples were then packed under vacuum and frozen at -18°C within a few hours from collection. A total of 30 animals were sampled (10 for each area), balanced by sex and age (all the animals were in the class subadults aging12-24 months).

**Table 2:** Morphological traits (mean±SD) of female (F) and male (M) wild boars included in the trial according to harvesting area (TC = Tyrrhenian Coast; MA = Maremma, CP = Central plains) and sex.

	Harvesting area					
	TC		CP		MA	
	F	M	F	M	F	M
N.	5	5	5	5	5	5
Age (months)	16.8±1.8	18.4±0.5	15.4±1.9	21.7±3.2	18.2±1.8	19.0±4.0
Bodyweight (kg)	36.2±7.1	38.2±9.6	37.2±9.8	45.5±14.5	34.2±10.1	36.1±7.0
Total length (cm)	108.4±6.4	105.2±6.6	111.5±10.9	113.0±9.9	104.2±11.4	103.8±6.1
Height at withers (cm)	62.5±3.3	63.8±4.5	66.6±6.2	67.0±5.2	63.4±5.8	61.9±3.0

The processing consisted in the separation of hairs from skin and derma and muscular elements from fat. Feces and other contaminants were removed from hair utilizing ultra-sound agitation in deionized water, shaken in a 2:1 mixture of methanol/chloroform for two hours and rinsed with deionized water. After drying (40°C, 48 h), duplicate hair samples from each animal were selected in order to verify repeatability of the analytical procedures [5]. After separation, tissues were cut into small pieces, placed in sanitized screw top vials and dried overnight [14] at a constant weight with the aid of a freeze drier. The dried pieces were homogenized with a suitable grinder and freeze-dried again. De-fatted muscles are not affected by possible alterations of the nitrogen signal owing to contamination by lipids. In fact, we checked that no nitrogen traces were present in the fat.

Afterwards, the fat-free dry mass and the lipid fractions (after solvent evaporation) were stored in an appropriate container in a vacuum desiccator until measurement [2]. The final count included: 30 samples of muscle, 30 samples of hair tip and 30 of hair base (proximal and distal, 10 mm sections from the hair’s end, respectively) and 30 of peri-renal fat. Although no direct measure of hair growth was performed, we estimated the period of growth according to Holà et al. [14].

### Stable isotope ratio analysis

All the prepared samples were admitted to a CF-IRMS system by means of a Dumas combustion elemental analyzer NA1500 (CARLO ERBA, Milan, Italy). The isotope ratio mass spectrometer was an Isoprime GV (Elementar Gmbh, Isoprime Ltd, Germany), tuned to analyze stable isotope ratios of carbon (^13^C/^12^C) and nitrogen (^15^N/^14^N), respectively. Isotope composition (δ) is then calculated as the deviation from the unit of the ratio of the isotope ratio of a sample to that of the international standard according to Farqhar et al. [22]:

$$\delta =\left[\left({\text{R}}_{\text{sample}}/{\text{R}}_{\text{standard}}\right)-\text{t}\right]$$

where R_sample_ and R_standard_ are respectively the isotope ratio of samples (^13^C/^12^C or ^15^N/^14^N) and of the international standard, i.e. Vienna Pee-Dee Belemnite (VPBD) for δ^13^C and atmospheric nitrogen for δ^15^N. The precision of isotopic determinations, expressed as S.D. of ten repetitions of the same gaseous specimen, was better than 0.01‰for both δ^13^C and δ^15^N. The measurements were anchored on the VPDB and atmospheric N_2_ scales by means of international standards by IAEA and USGG. The ranges of standards used for calibration were from -31.8 to +2.0‰ for δ^13^C and from -30.4 to + 375.3‰ for δ^15^N.

### Statistical analysis

The isotopic compositions were statistically analyzed using the General Linear Model Procedure of Statistica 10 (StatSoft Inc., USA). Through a full factorial model, the effects of the area of collection (*A*; TC, MA or CP), animal sex (*Sx*; male M or female F) plus the area × sex interaction on the C and N isotopic signatures in different sample types (muscle, hair tip, hair base, fat) were evaluated. In order to test the effects of the harvesting area (A) and the sample type (muscle, hair tips or hair base and peri-renal fat) on C and N isotope compositions separately, another full factorial model was used. The body weight (*BW*; in kilograms) was tested as a covariate in both models. However, the latter, being not significant, was excluded from further analyses. The comparison between the means was performed using the Tukey test. The relationship of the isotopic signature for C and N in different sample types was also investigated using Pearson’s product moment correlation. The significance was declared for *P*<0.05.

## Results

Table 3 reports reference values of δ^13^C and δ^15^N inwild boar hair (tip and base), muscle and fat, as these values are only available for hair in corn fed animals [14].

**Table 3:** Effect of the area of harvest and gender on δ^15^N and δ^13^C (mean±SD) in muscle, hair tip, hair base, and fat of free-living wild boar.

	Area (*A*)			Sex (*Sx*)		*P*-level		
	TC	CP	MA	M	F	*A*	*Sx*	*A×Sx*
δ^13^C (‰)								
Muscle	-25.1±1.0^b^	-23.5±1.3^a^	-24.3±1.2^ab^	-24.4±0.3	-24.7±1.4	<0.05	ns	ns
Hairtip	-23.8±1.0^B^	-21.6±1.1^A^	-22.7±1.4^AB^	-22.7±0.7	-23.3±0.7	<0.05	ns	ns
Hair base	-23.2±1.3^b^	-21.0±2.2^a^	-22.3±1.8^ab^	-23.1±0.2	-21.5±2.3	<0.05	ns	ns
Fat	-29.2±1.1^b^	-28.1±0.9^a^	-28.9±1.2^ab^	-29.9±0.8	-29.1±1.3	<0.05	ns	ns
δ^15^N (‰)								
Muscle	2.9±1.0^ABb^	4.9±1.5^Aa^	3.3±1.8^Bc^	1.3±1.0	3.4±2.7	<0.001	ns	<0.05
Hairtip	2.9±1.3^AB^	4.4±1.5^A^	3.1±1.8^B^	1.2±0.6	2.9±2.4	<0.01	ns	ns
Hair base	2.6±1.5^AB^	4.5±1.5^A^	2.9±1.9^B^	1.0±0.9	2.8±2.5	<0.01	ns	ns

Superscripts: capital letters within the same line denote significant differences at *P*>0.01; lower case letters denote significant differences at *P*<0.05; ns = *P*≥0.05; TC: Tyrrhenian Coast, MA: Maremma, CP: Central plains.

The outputs of the GLM procedure showed that area is the main factor capable of discriminating data (multivariate test; Wilks' lambda: 0.058, *P*<0.01; Roy's largest root: 5.505, *P*<0.01). In all the tissues, an enrichment of δ^13^C in CP area was observed in comparison to the other areas. The same effect was observed for δ^15^N, which also showed a significant enrichment in CP area. In detail, we observed that muscle, hair and fat were less depleted of δ^13^C in CP area in comparison to TC (Table 3). No differences were observed between sexes and no significant area ×sex interactions were found, except in the muscle for δ^15^N. In the light of the null effect of sex on the isotopic signatures of the tissue samples, the influence on the isotopic compositions of the sample type was tested along with its interaction with the area of harvesting. Sample type resulted highly significant with regard to the isotopic signature of carbon but not for δ^15^N (Table 4).

**Table 4:** C and N isotopic compositions (mean±SD) of muscle, hair tip, hair base, and fat of free-living wild boar harvested in different areas.

	Sample type (tissue and/or sampling position)				*P*-level
δ^13^C	Muscle	Hair tip	Hair base	Fat	
TC	-25.0±0.9^Ab^	-23.7±1.1^Aab^	-23.1±1.2^Aa^	-29.2±1.1^B^	< 0.001
CP	-23.5±1.3^B^	-21.6±1.1^AB^	-21.0±2.2^A^	-28.1±0.9^C^	
MA	-24.4±0.3^A^	-22.7±0.7^A^	-23.1±0.2^A^	-29.9±0.8^B^	
δ^15^N					
TC	-25.0±0.9	-23.7±1.1	-23.1±1.2		ns
CP	-23.5±1.3	-21.6±1.1	-21.0±2.2		
MA	-24.4±0.3	-22.7±0.7	-23.1±0.2		

Superscripts: capital letters within the same line denote significant differences between sample types at *P*>0.01; lower case letters denote significant differences between sample types at *P*<0.05; ns = *P*≥0.05; (TC: Tyrrhenian Coast, MA: Maremma, CP: Central plains)

No differences were recorded between the basal and tip portion of the hair (*P* = 0.22 and *P* = 0.24 as far as δ^13^C and δ^15^N, respectively) (Table 4). Overall, no differences were recorded for δ^13^C and δ^15^N between the two portions of hair within gender. The only exception concerned the males of CP area (where corn is provided) that showed a significant (*P*<0.05) higher δ^13^C in the basal portion (unreported results).

Overall, the individual isotopic compositions were found to be highly correlated among different tissues (Table 5). It is worth noting the high, positive, and significant correlation between δ^15^N values in the basal portion of hair and in the muscle samples as well as the correlation between the values of the two hair portions (Table 5).

**Table 5:** Correlations among δ^13^C and δ^15^N in different sample types of free-living wild boar harvested in different areas.

	Muscle δ^13^C	Hair tip δ^13^C	Hair base δ^13^C	Fat δ^13^C	Muscle δ^15^N	Hair tip δ^15^N
Hair tip δ^13^C	0.450*					
Hair base δ^13^C	0.212	0.607**				
Fat δ^13^C	0.491*	0.339	0.542**			
Muscle δ^15^N	0.539**	0.245	0.380	0.674***		
Hair tip δ^15^N	0.545**	0.352	0.277	0.641**	0.873***	
Hair base δ^15^N	0.494*	0.324	0.312	0.636**	0.933***	0.953***

* *P*<0.05.

** *P*<0.01

*** *P*<0.001.

## Discussion

The mean values of δ^15^N and δ^13^C in the animal tissues provide information on both the trophic level and/or the feeding regime. This is particularly relevant for reconstructing the diet of free-living animals. Biological fixation of N determines δ^15^N values close to 0‰ and generally increases with an increase in the trophic level [23]. In fact, herbivores show higher δ^15^N values than those in their production source, i.e. their diet. The phenomenon is magnified along the trophic network and carnivores invariably show higher values than their preys. Due to metabolic isotopic fractionations, an animal generally presents an enrichment between 3–5 ‰ of δ^15^N higher when compared to its diet [16,24].

Analysing values of isotopic compositions can provide qualitative information on wild boar nutrition. Nevertheless, a precise interpretation can be difficult or even impossible due to the feeding habits of free living wild boar [25–27]. References to this purpose are provided by other authors [14] who tested a corn-based diet. Their results should be considered as a milestone and an extreme boundary because a crop-based diet was used in pen-reared animals. On the other hand, the opposite extreme of a non-crop feed-based diet can be obtained only in natural conditions and where the availability of crops can be reasonably excluded. In our study, harvesting animals in the autumn-winter period was chosen to allow the feeding on acorns and other natural crops available in the tested areas [28]. Wild boar diet varies seasonally, being based on agricultural plants in summer and spring and on non-agricultural plants, acorns and animal matter in winter [15,25]. This shift can be easily observed because grasslands are visited by wild boar mainly in winter whilst cereals are damaged from the milky stage to ripeness [29].

In the present study, the areas indicate remarkable differences and we can consider the three populations of wild boar show different feeding habits due to the availability and/or administration of C4 or C3 foods. It is important to highlight that the isotopic compositions reflect the ingested feed [9,30] but also the metabolism of the organism and, in particular, of the single tissue or organ [12]. In this figure, muscles reflect recent dietary intake, because of their high metabolic activity and turnover; on the contrary, hair is a metabolically inert tissue, reflecting the diet consumed during the period of growth [14]. However, it is difficult to establish if the protein sources are of plant or of animal origin [16].

In this study, the hair δ^13^C and δ^15^N values result similar to those ofthe muscle (Table 4). This finding can be referred to a stable diet in the short term, as suggested by the values of the base portion of the hair and, on a time scale of a few months, by the values of the hair tip portion. Estimating a hair growth rate of about 1 mm/day, a period correspondent to the analyzed hair length is about 2–3 months. Thus, no significant differences in δ^13^C and δ^15^N comparing the lower and higher sections of the specimens (Table 4) indicate scarce variability of the diet in the short and long term (months). Changes in δ values allow inference about seasonal ecosystem changes or may identify differences between production systems [5]. However, isotopic signals from different organs or tissues could evidence variations in the diet. In temperate regions, common δ^13^C values of plants eaten by herbivorous animals of about -25±2‰ [31] are in reasonable agreement with the mean δ^13^C values that we observed inboth muscle and hair of wild boar. However, in CP, we found on the hair base a markedly enriched δ^13^C value (-21.0‰; Tab 3), most likely related to the supplementary feeding with corn.

The obtained δ^13^C results highlight the effect of corn spreading carried out in the CP area in contrast to the other two areas. In the TC and MA areas the nutritional requirements of wild boars are satisfied predominantly by plant matter (90–99% of ingestion), with a relatively poor supplement of animal matter (1–10%), according to seasonal availability [27]. In the Mediterranean biomes, the most commonly ingested part is the underground portion of the plants, such as roots and bulbs (ingested mainly after rooting activity), followed by green parts and fruits (fleshy or Mediterranean scrub fruit). The lowest proportion of ingested food is animal matter in the form of earthworms, terrestrial arthropods and carcasses, insect larvae and birds (brooding or moulting) [26,27].

In the present study, the analysis of δ^15^N values indicates remarkable differences among the areas. This is especially due to the CP area, where corn was spread to attract and familiarize the animals with the hunting area. These differences are in accordance with the different management practices and/or locally different environments [16] as previously observed in the same area for heavy metals [32]. Particularly, the relatively high values in δ^15^N found in the wild boar specimens from CP could indicate a more intensive agricultural management than in the other two sampling sites. The intermediate δ^15^N values observed in TC are likely explained by the high wilderness of this area, which is characterised by a massive coverage of Mediterranean forest biocenosis and more closed N cyclings. Finally, the relatively arid conditions of MA and its extensive agricultural management are both likely explanations of the correspondent low δ^15^N values in wild boar tissues.

## Conclusions

The research demonstrates that both carbon and nitrogen isotope compositions in free-living wild boar tissues are affected by the sampling area. According to feeding habits of the species and management regimes of the areas, the observed differences of δ^13^C and δ^15^N are based on the specific feeding regime, with particular evidence related to the use of corn. The research also allowed the establishment of stable carbon and nitrogen isotope ratios in hair, fat and muscles of free-living wild boar, confirming the correlation between hair and muscle.

Analysing other samples, like teeth and bone collagen, together with a detailed characterization of vegetation and of land use, could provide further isotopic assessments (strontium, sulfur and oxygen) in order to deepen investigation about the feeding behavior of wild boar.
